# Major biogeographic barriers in eastern Australia have shaped the population structure of widely distributed *Eucalyptus moluccana* and its putative subspecies

**DOI:** 10.1002/ece3.8169

**Published:** 2021-09-30

**Authors:** Lluvia Flores‐Rentería, Paul D. Rymer, Niveditha Ramadoss, Markus Riegler

**Affiliations:** ^1^ Department of Biology San Diego State University San Diego CA USA; ^2^ Hawkesbury Institute for the Environment Western Sydney University Penrith NSW Australia

**Keywords:** Eastern Australia, *Eucalyptus moluccana*, geographic barriers, grey box, subspecies formation

## Abstract

We have investigated the impact of recognized biogeographic barriers on genetic differentiation of grey box (*Eucalyptus moluccana*), a common and widespread tree species of the family Myrtaceae in eastern Australian woodlands, and its previously proposed four subspecies *moluccana*, *pedicellata*, *queenslandica*, and *crassifolia*. A range of phylogeographic analyses were conducted to examine the population genetic differentiation and subspecies genetic structure in *E. moluccana* in relation to biogeographic barriers. Slow evolving markers uncovering long term processes (chloroplast DNA) were used to generate a haplotype network and infer phylogeographic barriers. Additionally, fast evolving, hypervariable markers (microsatellites) were used to estimate demographic processes and genetic structure among five geographic regions (29 populations) across the entire distribution of *E. moluccana*. Morphological features of seedlings, such as leaf and stem traits, were assessed to evaluate population clusters and test differentiation of the putative subspecies. Haplotype network analysis revealed twenty chloroplast haplotypes with a main haplotype in a central position shared by individuals belonging to the regions containing the four putative subspecies. Microsatellite analysis detected the genetic structure between Queensland (QLD) and New South Wales (NSW) populations, consistent with the McPherson Range barrier, an east‐west spur of the Great Dividing Range. The substructure was detected within QLD and NSW in line with other barriers in eastern Australia. The morphological analyses supported differentiation between QLD and NSW populations, with no difference within QLD, yet some differentiation within NSW populations. Our molecular and morphological analyses provide evidence that several geographic barriers in eastern Australia, including the Burdekin Gap and the McPherson Range have contributed to the genetic structure of *E. moluccana*. Genetic differentiation among *E. moluccana* populations supports the recognition of some but not all the four previously proposed subspecies, with *crassifolia* being the most differentiated.

## Graphical abstract

Our molecular and morphological analyses provide evidence that several geographic barriers in eastern Australia, including the Burdekin Gap, the McPherson Range and the Sydney Sandstone Belt, have contributed to the genetic structure of *E. moluccana* and the formation of the four subspecies.

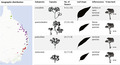

**Significant Statement**

Landscapes include barriers to dispersal and gene flow, and their effects on species vary over evolutionary time. We have assessed the impact of known barriers on the ecologically important woodland tree, *Eucalyptus moluccana*, across its wide distribution in eastern Australia. Using genetic and morphological analyses, we found that geographic and biological barriers have shaped its population genetic structure and contributed to the formation of subspecies. However, admixture among four proposed subspecies was detected, suggesting recent divergence. Our findings are important for conservation and revegetation efforts as this species occurs across a large climatic gradient with potential for local climatic adaptation.

## 1 INTRODUCTION

In contrast to the generally low topographic relief of Australia, eastern Australia has a moderate elevation profile provided by the Great Dividing Range (GDR), which was formed through several periods of tectonic uplift over the past 70 Myr (Frakes et al., 1987; Keast, 1981; Taylor, 1994). This mountain system spans over 3,500 km from south to north. Despite its relatively moderate elevation (average 1,000–1,300 m, maximum 2,300 m), it provides substantial elevational, climatic and environmental variation (Keast, 1981; Taylor, 1994), which may influence the ability of species to expand or contract their ranges in response to climatic oscillations (Byrne, 2008) and thereby affect their genetic structure and ongoing speciation processes. Palaeoclimatic studies indicate that the extent and composition of the vegetation along the GDR has fluctuated dramatically over the last 20 to 10 Myr, with a general transition from rainforests to drier environments with sclerophyllous vegetation (Byrne, 2008; Martin, 2006; Williams, 2000). Australia was not subject to major glaciations as seen across much of the northern hemisphere—only 0.5% of Australia was glaciated during the Pleistocene (Williams, 2000, 2001). However, Australia became progressively drier, culminating in extreme aridification during glacial cycles (Williams, 2000), setting the stage for the evolution of arid‐adapted biota. Cool–dry to warm–wet climatic fluctuations that commenced during the Pliocene, intensified throughout the Pleistocene and led to the repeated expansion and contraction of mesic habitats in eastern Australia and their regular encroachment by drier habitats (Byrne et al., 2008; White, 1994). Currently, the once widespread rainforest and mesic forest vegetation is restricted to small, scattered remnants within large areas of dry sclerophyll woodlands and open forests of eastern Australia (Byrne, 2008; White, 1994).

The evolutionary history of species resident in eastern Australia has been influenced by physical and environmental barriers, which led to genetic divergence and, in some cases, speciation of allopatric populations (Byrne et al., 2008; Chapple et al., 2011). A well‐studied barrier is the Black Mountain Corridor situated within the Wet Tropics of northern Queensland (QLD, Rossetto et al., 2007, 2009). Several studies of a wide range of rainforest taxa across this region revealed largely concordant patterns of genetic divergence across this dry habitat barrier (Bryant & Krosch, 2016; Chapple et al., 2011). At least nine other biogeographic barriers have been identified in eastern Australia, such as dry habitat barriers (Burdekin Gap, St Lawrence Gap, Hunter Valley), mountain ranges that act as topographic barriers (McPherson Range, Southern Table, and Highlands of New South Wales [NSW]), plains surrounding disjunct inland mountains (Kroombit Tops), sea straits (Bass Strait), and former marine basins (Gippsland Basin, Murray Basin) (Bryant & Krosch, 2016; Chapple et al., 2011). These barriers, in concert with climatic oscillations, have generated the high levels of biodiversity evident in eastern Australia (Bryant & Krosch, 2016).

Many of these barriers constitute dry habitat breaks of mesic environments and thereby have led to genetic structure in mesic biota. For example, several barriers dominated by open canopy forest communities (e.g., eucalypt woodlands) have influenced the genetic structure of widely distributed animals such as the sedge frog (James & Moritz, 2000), an agamid lizard (Edwards & Melville, 2010), and the delicate skink (Chapple et al., 2011). Other animals with more reduced distributions have also been influenced by one or more of these barriers (reviewed in Bryant & Krosch, 2016; Chapple et al., 2011). How these barriers have shaped the genetic structure of plants is relatively less known and has been investigated for a few genera such as *Eucalyptus* (Jones et al., 2006; Shepherd et al., 2010), *Lomatia* (Milner et al., 2012, 2013, 2015), and *Telopea* (Rossetto et al., 2012). Most of these studies included two or more species, each of which occupy different geographic regions, but not the entire GDR, or large sections thereof; an exception is *Eucalyptus grandis*, a widespread and dominant tree species of wet sclerophyll forests which displays some differentiation across its range in central and northern regions of eastern Australia (Jones et al., 2006).

Dry habitats may not constitute barriers to taxa of open forest communities. For example, the dry habitat barrier of the Hunter Valley has played an important role in structuring several *Lomatia* (e.g., Milner et al., 2012) but does not seem to have affected the structure of *Corymbia maculata* found in open forest communities (Shepherd et al., 2012). Therefore, although dry habitat barriers such as the Hunter Valley, St Lawrence Gap, and the Burdekin Gap have affected populations of rainforest taxa that have become separated from other populations in increasingly disconnected rainforest refugia (Bryant & Krosch, 2016), it is less clear whether they also affected dry sclerophyllous vegetation communities. For example, dry habitat species may have experienced reduced gene flow due to interspersed mesic communities.

Despite the dominance and diversity of *Eucalyptus* in eastern Australia (ABARES, 2013), relatively few studies have evaluated the effect of biogeographic barriers on its population structures and speciation processes (Jones et al., 2006, 2013; Shepherd & Raymond, 2010), showing strong association between population genetics and geography (Pollock et al., 2013). Here, we studied the influence of eastern Australian biogeographic barriers on the population genetic differentiation and subspecies genetic structure of a *Eucalyptus* species that is common in dry grassy woodlands, the grey box, *Eucalyptus moluccana* Roxburgh (1832). This species is ecologically important providing habitat and food for bees and native insects, wild animals such as flying foxes, black‐chinned honeyeaters, greater gliders, vulnerable species like koalas, and critically endangered species such as the swift parrot and the regent honeyeater (Kennedy & Tzaros 2005; Martin et al., 2003; Oliver 2000; Smith et al., 2007). Although *E. moluccana* is a dominant species on dry coastal plains, open valleys and ranges, it has lost a large portion of its original habitat due to agriculture and urban development. In some areas this loss has reached more than 94% since European settlement, e.g., in the Cumberland Plain (Benson & Howell, 1990). It has a wide latitudinal distribution, ranging from the Jervis Bay area (NSW) to the Atherton Tableland (QLD), spanning across most of the barriers in eastern Australia, allowing us to test the effect of biogeographic barriers in eastern Australia on the structuring of dry sclerophyllous vegetation. This is particularly important because some studies have suggested a barrier might counterintuitively be responsible for structuring a species even when the species occurs within the region of the barrier (Bryant & Krosch, 2016), and other undescribed barriers should be invoked. This issue arises because most of the biogeographic barriers have been proposed for mesic species, and limited information is available to determine barriers structuring species in drier environments.

**FIGURE 1:**
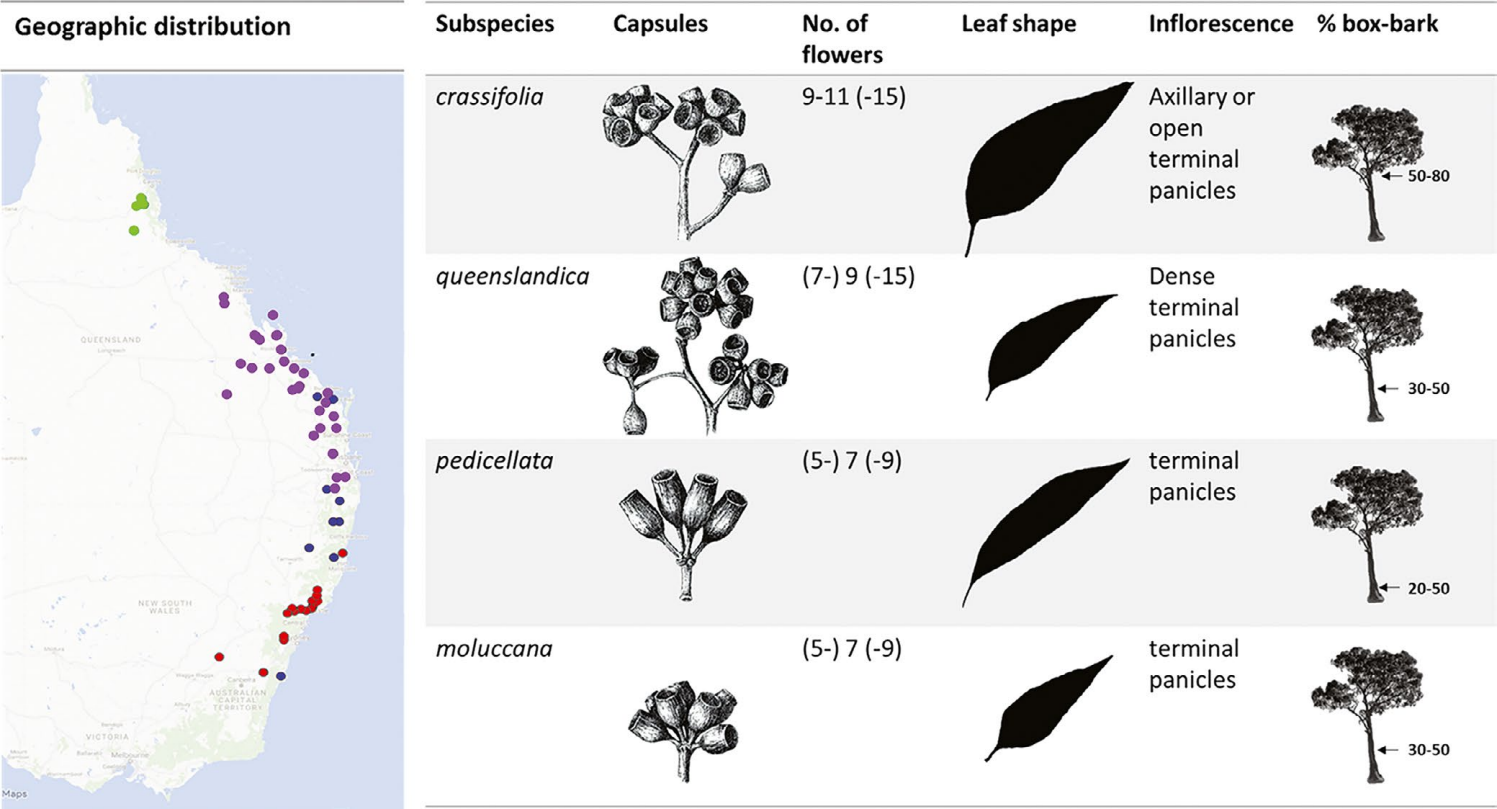
Map of the geographic distribution and morphological features of four putative subspecies in *Eucalyptus moluccana* as per Gillison (1976). Dots on the map indicate populations that were characterized and assigned by Gillison (1976) to four putative subspecies: *crassifolia* (green; capsule: 4.5–5.5 × 4.0–4.5 mm, juvenile leaf: 7–16.5 × 4.5–10 cm), *queenslandica* (purple; capsule: 4.0–5.8 × 3.5–4.8 mm, juvenile leaf: 8–17 × 2.5–8.5 cm), *pedicellata* (blue; capsule: 4–11 × 3.5–6.5 mm, juvenile leaf: 8–13 × 4–7 cm) and *moluccana* (red; capsule: 4.5–8.5 × 4–6 mm, juvenile leaf: 6–12 × 4.5–2 cm). Morphological features that distinguish these subspecies are capsule shape, pedicel, number of flowers per umbellaster, leaf shape, inflorescence, and proportion of box‐type bark out of tree height. Drawings modified from Gillison (1976)

Interestingly, it has previously been suggested that *E. moluccana* is subdivided into four subspecies (subspecies *moluccana*, subspecies *pedicellata*, subspecies *queenslandica*, and subspecies *crassifolia*) based on morphological differences, including the number of flowers per peduncle, inflorescence type, capsule shape, presence of capsule pedicel, leaf shape, and proportion of box‐type bark (Figure 1; Gillison, 1976). These proposed four subspecies have different latitudinal distributions, with subspecies *moluccana* occupying the southern distribution from the Sydney Basin to the NSW Mid North Coast, overlapping with subspecies *pedicellata* in northern NSW, which in turn overlaps with subspecies *queenslandica* in southern QLD. The latter is distributed up to Mackay, with a large gap between Mackay and the southern limit of the subspecies *crassifolia* (Figure 1). However, no previous genetic evidence has been established to test this differentiation into four subspecies. Additionally, Gillison (1976) analyzed leaf shape in juvenile, intermediate, and mature trees. As eucalypts are heteroblastic with seedlings expressing different leaf morphology than mature trees and juvenile leaves being more taxonomically informative, separating among close relatives (Bean, 2009; Flores‐Rentería et al., 2017; Ladiges et al., 1981; Rutherford et al., 2017), leaf morphometrics of seedlings might be more conclusive than leaf morphometrics of mature leaves. Leaf morphology can be plastic; therefore, growing the plants from different localities in the same environment is important to separate the plasticity due to environmental factors.

In this study, we (a) evaluated whether biogeographic barriers have played a role in the genetic structure of *E. moluccana*, (b) identified which particular geographic and habitat barriers contributed to the genetic structure, and (c) assessed whether the suggested subspecies are isolated by these barriers. We hypothesized that there is genetic differentiation among the subspecies of *E. moluccana* and that this differentiation might be due to biogeographic barriers reducing connectivity among subspecies. Moreover, if this genetic differentiation is strong, we would be able to see morphological differentiation of seedlings (in particular juvenile leaves) among the subspecies grown in the same environment.

## 2 MATERIALS AND METHODS

### 2.1 Species distribution and barrier identification

*Eucalyptus moluccana* Roxburgh, 1832 (syn. *E. hemiphloia* F. Muell. 1866), commonly known as the grey box or gum‐topped box, is a member of the eastern grey boxes (subgenus Symphyomyrtus; section Adnataria; series Buxeales) (Flores‐Rentería et al., 2017). It is a medium‐sized to occasionally tall tree with rough, persistent bark on the lower trunk, shedding above to leave a smooth whitish or light grey, sometimes shiny surface. Grey box forms part of grassy woodland communities and is widespread across eastern Australia from 17° to 35°S. Lat., with a range of 535,807 km² (nswnichefinder.net). All reliable records of *E. moluccana* in the Atlas of Living Australia (ALA, https://www.ala.org.au/) were plotted on a map using the ALA interface. Excluded were very old records and records lacking coordinate decimals and records of specimens that were not within woodlands or woodland fragments. Then records of occurrence for *E. moluccana* were used to determine whether this species occurs within regions defined as habitat barriers.

### 2.2 Collections

**FIGURE 2:**
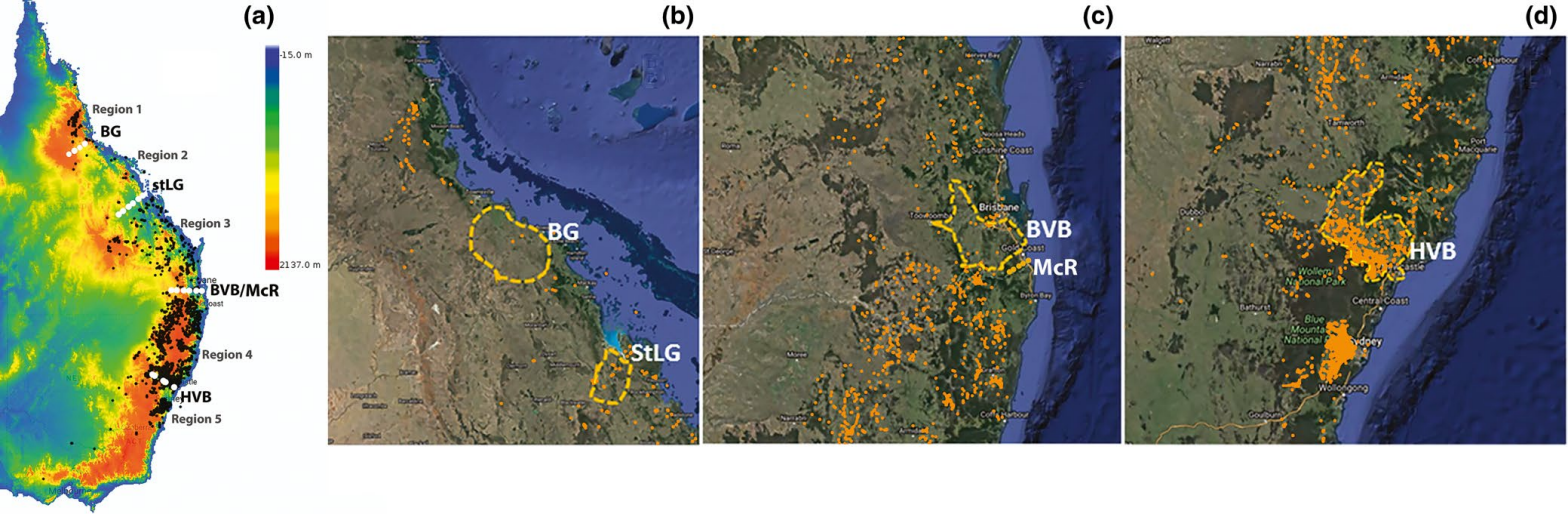
(a) Occurrence of *Eucalyptus moluccana* based on Atlas of Living Australia is represented by black dots, four biogeographic barriers identified on eastern Australia (Bryant and Krosch (2016) are represented by white dotted lines, and the Great Dividing Range is represented by higher altitudes shown in orange along eastern Australia. Occurrence of *E. moluccana* is shown in orange across the following biogeographic regions: (b) Burdekin Gap (BG), and St Lawrence Gap (StLG), (c) Brisbane Valley Barrier (BVB)/McPherson Range (McR), and (d) Hunter Valley (HVB) to the north of the Sydney Sandstone Belt

Plant material was collected throughout the entire distribution of *E. moluccana*, from localities at least 50 km apart from each other. In NSW, the distribution of *E. moluccana* is clustered in open grassy woodlands found in the plains and valleys; however, in QLD, populations are scattered, with substantial gaps within the northern part of its range (Figure 2a). Samples were collected from 29 locations over five regions (northern, central, southeastern QLD, northern NSW, and Sydney Basin) encompassing four biogeographic barriers according to Bryant and Krosch (2016). These populations came from regions harboring the previously proposed four subspecies, subspecies *moluccana*, subspecies *pedicellata*, subspecies *queenslandica*, and subspecies *crassifolia* (Figure 1; Gillison, 1976) (Figure 2a‐d, Table 1).

**TABLE 1:** *Eucalyptus moluccana* populations and subspecies included in this study

Population/location	Subspecies	Sample size genetic analysis	Sample size seedling morphology	Latitude	Longitude	Altitude	Region^a^
Mount Garnet	*crassifolia*	14	17	−17.7167	145.0333	725	1
Tumoulin	*crassifolia*	10	16	−17.5333	145.4333	970	1
Gunnawarra^b^	*crassifolia*	1	12	−18.1667	145.2833	710	1
Crediton	*queenslandica*	23	30	−21.2500	148.4833	650	2
Calliope	*queenslandica*	17	20	−23.9500	151.1000	40	3
Biloela	*queenslandica*	17	16	−24.2500	150.5333	280	3
Coominglah	*queenslandica*	25	35	−24.8167	150.9667	450	3
Tiaro	*queenslandica*	14	20	−25.7500	152.5833	40	3
Running Creek	*queenslandica*	15	20	−25.9000	152.3167	80	3
Wondai	*queenslandica*	3	6	−26.3167	151.9000	350	3
Ballon	*queenslandica*	3	6	−26.4500	150.8167	325	3
Unumgar State Forest	*pedicellata*	**14**	3	−28.3924	152.7193	240	4
Sunnyside	*pedicellata*	2	5	−29.0078	151.9600	830	4
Bom State Forest	*pedicellata*	**12**	3	−29.7390	152.9654	70	4
Taree	*pedicellata*	**15**	0	−31.8600	152.4804	29	4
Wallaroo	*moluccana*	**2**	0	−32.6382	151.8731	91	4
Singleton	*moluccana*	**9**	0	−32.6422	151.2321	85	4
Belford	*moluccana*	**16**	0	−32.7146	151.2915	69	4
Millfield	*moluccana*	**16**	0	−32.8990	151.2430	147	4
Putty	*moluccana*	**6**	0	−32.9304	150.6989	238	5
Scheyville	*moluccana*	3	8	−33.6083	150.8981	55	5
Knudsen Reserve	*moluccana*	**2**	0	−33.6850	150.8495	15	5
Richmond	*moluccana*	3	6	−33.6317	150.7794	20	5
Liberty Grove	*moluccana*	**6**	4	−33.8495	151.0795	6	5
Bellevue Reserve	*moluccana*	**10**	0	−33.9083	150.9989	51	5
Luddenham	*moluccana*	3	6	−34.8808	150.6894	110	5
Ingleburn	*moluccana*	3	6	−34.0103	150.8728	64	5
Mount Annan Botanical Garden	*moluccana*	6	8	−34.0662	150.7739	165	5
Nowra	*moluccana*	**9**	0	−34.9673	150.5936	44	5

Note

Subspecies were determined based on the distribution and features described in Gillison (1976). Numbers in bold represent the sample size of mature trees included in the genetic analysis. Numbers not in bold represent the sample size of seedlings included in the genetic analysis (one seedling per maternal tree).

^a^ 1 Northern Queensland, 2 Central Queensland, 3 South East Queensland, 4 Northern NSW, 5 Sydney Basin.

^b^ Samples from Gunawarra were excluded because they failed to amplify some microsatellites.

Two sampling approaches were done for plant collection. We collected leaves from mature individuals from 12 localities in the field. Leaves were collected from approximately 20 mature trees at each locality, with a minimum distance of 20 m between sampled trees. Trees were GPS‐mapped. To supplement the field collections, seed collected from open pollinated trees (>20 m apart) was obtained from the NSW Seed Bank (The Royal Botanic Gardens & Domain Trust) and The Australian Tree Seed Centre (CSIRO). Seed collection included a total of 17 localities. Seeds were germinated as specified before, and leaves were collected from seedlings. For our genetic analyses, a single seedling was sampled from each mother tree.

### 2.3 Seed germination and seedling morphometrics

*Eucalyptus* species have heteroblastic leaves (leaf type depends on tree age), and juvenile leaf morphology is a taxonomically informative feature (Bean, 2009; Flores‐Rentería et al., 2017; Ladiges et al., 1981; Rutherford et al., 2017). Seeds from all subspecies (localities and sample sizes are found in Table 1) were planted in a soil mix, watered daily, and maintained in a glasshouse with constant relative humidity (70%) and temperature (26°C) for 6 months and then transferred to a polytunnel. Plants were rotated each month over 18 months. Morphometric analysis was performed on fully expanded juvenile leaves and stem traits of six‐month‐old seedlings. Three leaves per plant were selected from the third, fourth and fifth nodes from the apical meristem. Digital vernier calipers were used to measure petiole length, leaf ratio (length blade:maximum width), stem diameter (average of two measurements), and height. These measurements were made when seedlings were 6 months old and then at 18 months old. A linear discriminant analysis of morphometric leaf characters of four subspecies of *E. moluccana* was performed in JMP (JMP statistical software, SAS, 2015) with subspecies identification following Gillison (1976) clustering as a prior method of grouping individuals.

### 2.4 DNA extraction

A modified CTAB (2%) protocol was followed (Doyle, 1987) to extract DNA from leaves of both two‐month seedlings and mature trees. DNA was visualized in agarose gel and normalized using nanodrop.

### 2.5 Chloroplast markers and haplotype network analysis

The intergenic region *psbA‐trnH* is among the most variable regions of the chloroplast in *E. moluccana* (Flores‐Rentería et al., 2017). Sequences for this chloroplast intergenic region were obtained using the methods of Flores‐Rentería et al. (2017). Additionally, sequences from a subset of seedlings already available were included (accession numbers KY596186 – KY596665). DNA of the chloroplast sequences was aligned using the multiple progressive alignment procedure of MUSCLE (Edgar, 2004), and manual corrections were done around indels or microsatellites. Minimum spanning haplotype networks (Bandelt et al., 1999) were constructed using the program PopART (Leigh & Bryant, 2015) to better visualize nonbifurcating relationships (multifurcations and reticulations) in chloroplast haplotypes (Posada & Crandall, 2001). DNAsp software was used to create the haplotype data file (Librado & Rozas, 2009). PopART software was used to infer the haplotype network by region (http://popart.otago.ac.nz).

### 2.6 Genetic structure using microsatellites

Initial testing of 65 microsatellites loci of *Eucalyptus* species was performed: 52 were designed based on the nuclear genomes available for *E. globulus*, *E. grandis*, *E. gunnii*, and *E. urophylla* (Brondani et al., 2002; Thamarus et al., unpublished; Yasodha et al., 2008; Faria et al., 2011; Myburg et al., 2014) and 13 developed for *E. leucoxylon* (Ottewell et al., 2005). All loci were sequenced in a subsample of the *E. moluccana* populations to check their variability and confirm that they were single copy homologous loci following the methods of Flores‐Rentería et al. (2017). Ten microsatellite loci of *E. moluccana* were amplified in a multiplex design (Table S1). Each specific forward primer was linked with one of the 5′ universal primer sequence tails and fluorescence‐labeled (Table S2; Flores‐Rentería et al., 2013; Flores‐Rentería & Whipple, 2011). PCR reactions were carried out using QIAGEN Multiplex PCR Kit following manufacturer's instructions, and amplicons were sized as in Flores‐Rentería and Krohn (2013).

We used ARLEQUIN v3.5 (Excoffier & Lischer, 2010) to test for deviation from the Hardy–Weinberg equilibrium (HWE) and to test for linkage disequilibrium among loci. MICRO‐CHECKER v.2.2.3 (van Oosterhout et al., 2004) was used to test for the presence of null alleles. Only ten nuclear variable markers that were highly reproducible and informative and conformed to patterns of neutrality were included in the analysis. These markers were BV682066, BV682112, BV682167, EL14, EL27, EU694398, EU699745, EU699755, GF101862, and GF101866 (Brondani et al., 2002; Faria et al., 2011; Ottewell et al., 2005; Thamarus et al., unpublished; Yasodha et al., 2008) (Table S1).

### 2.7 Structure analysis

Structure 2.2 (Pritchard et al., 2000), which involves posterior probability of the data for a given K, Pr(X|K), was used to cluster individuals into a number of population groups (K). K was determined following the admixture model with correlated alleles, with a K of 1 to 10. Twenty independent runs of 1,000,000 Markov Chain Monte Carlo generations and 100,000 generations of burn‐in were used for estimating each value of K. The optimal K value was determined by an ad hoc statistic, ΔK (Earl & VonHoldt, 2012) and compared with the mean likelihood values (http://taylor0.biology.ucla.edu/structureHarvester/ access date 10 May 2021). The number of Ks in the dataset was evaluated by using ΔK values estimated with the software STRUCTURE Harvester. The software CLUMPP 1.1 was used to find optimal alignments of independent runs (Jakobsson & Rosenberg, 2007), and the output was used directly as input to DISTRUCT 1.1, a program for cluster visualization (Rosenberg, 2004). Substructuring was done when appropriate as recommended by Janes et al. (2017). We complemented the STRUCTURE analysis with a discriminant analysis of principal components (DAPC) (Jombart, 2008). DAPC was used to plot clusters of genotypes. The absence of any assumption about the underlying population genetics model, in particular concerning the Hardy–Weinberg equilibrium or linkage equilibrium, is one of the main assets of DAPC (Jombart et al., 2010). DAPC was used to identify and describe clusters of genetically related individuals, as implemented in the R package Adegenet 2.0 (Jombart, 2008; Jombart & Ahmed, 2011). To identify the optimal number of clusters, the data are transformed using PCA, and k‐means was run sequentially with increasing values of k. An optimization to select the appropriate number of PC was done using a cross‐validation; this method uses a range of PC retention numbers, each cross‐validated 50 times (90%:10% training: validation stratified splits) so that it minimizes the mean squared error of reclassification. The different clustering solutions were compared using the Bayesian Information Criterion (BIC), and the lowest BIC was selected as the optimal clustering solution (Jombart & Collins, 2015). A PCoA analysis was performed in GenAlex v6.503 (Peakall & Smouse, 2012) to further compare clustering patterns. The analysis of molecular variance (AMOVA) were calculated using GenAlex v6.503 (Peakall & Smouse, 2012). The genetic variance was partitioned within and among the groups identified by molecular‐diversity and population‐structure analyses. To test isolation by distance (IBD) in our species, a Mantel test was performed to determine the correlation between genetic distance and geographic distance matrices calculated among individuals using 999 permutations at 5% nominal level in GenAlex v6.503 (Peakall & Smouse, 2012).

## 3 RESULTS

### 3.1 Recognized barriers along *E. moluccana* distribution

Following Bryant and Krosch (2016), wet/dry habitat barriers encompassed within the distribution of *E. moluccana* (Figure 2a) are (from north to south): (a) Burdekin Gap (BG) and (b) St Lawrence Gap (StLG, Figure 2b, both dry habitat barriers), (c) Brisbane Valley Barrier (BVB, Figure 2c) to the north of the Main/McPherson/Border Ranges (McR) and (d) Hunter Valley (HVB, a dry habitat barrier, Figure 2d) forming five disjunct regions. Both the Brisbane Valley and Hunter Valley have *E. moluccana* populations, the Hunter Valley in high density. Moreover, *E. moluccana* does not seem to grow in the sandstone belt formed by the Blue Mountains and the Hornsby Plateau surrounding the Sydney region. Because there is a continuum of *E. moluccana* from the Hunter Valley to the north, samples collected from the Hunter Valley were grouped in Region 4.

### 3.2 Morphometrics

**FIGURE 3:**
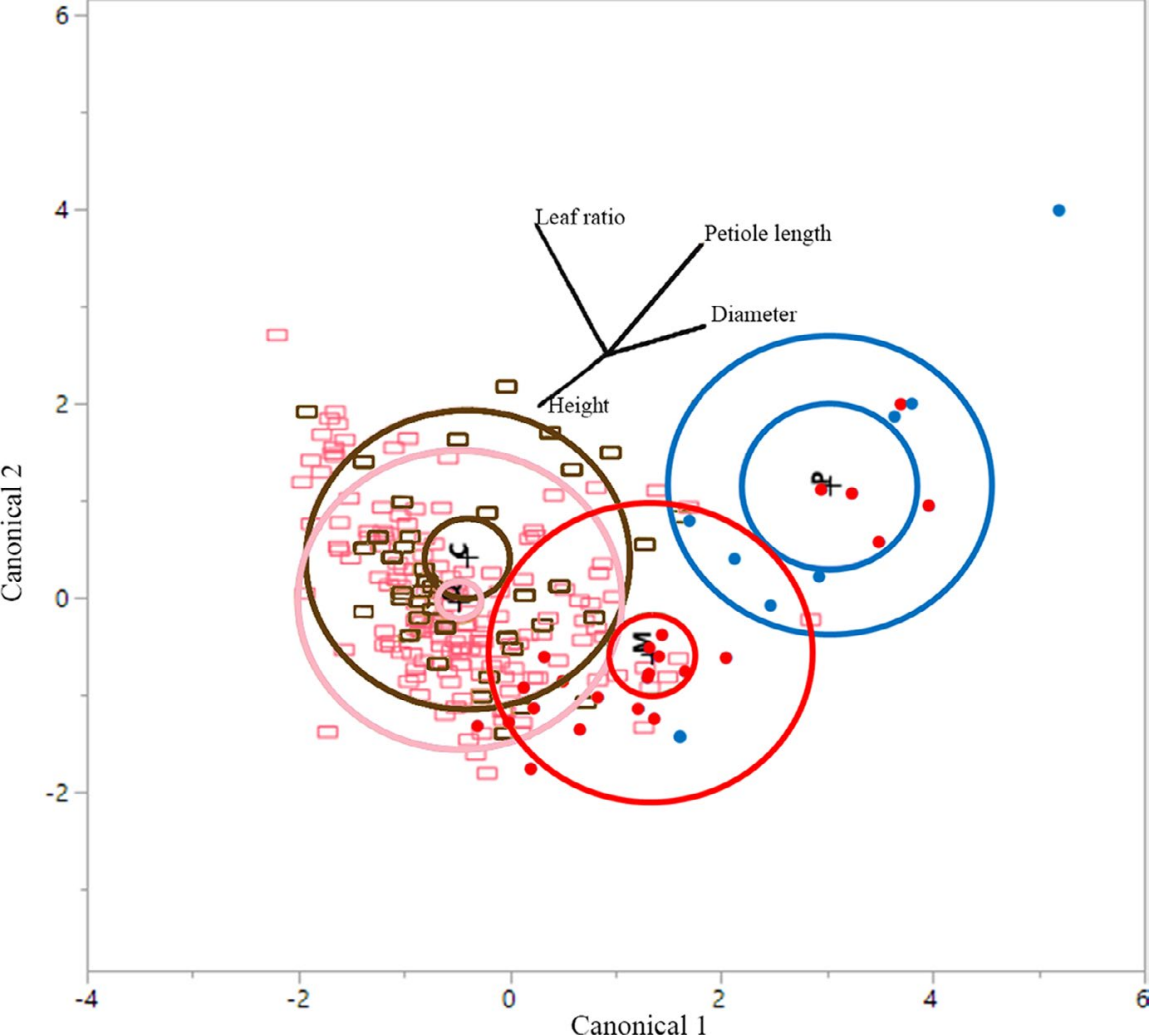
Canonical analysis of morphological features of leaves of six‐month old seedlings. Analyzed morphological features include leaf ratio, petiole length, stem diameter, and height. *Eucalyptus moluccana* putative subspecies *crassifolia* (C, brown open squares), *queenslandica* (Q, pink open squares), *pedicellata* (P, blue dots), and *moluccana* (M, red dots). Internal circle depicts the 95% confidence region for the means on the canonical variables of the group and the external circle denotes the space of the first two canonical variables and contains approximately 50% of the observations

At six months, seedlings showed some separation by leaf ratio between northern (QLD) and southern (NSW) populations, representing putative northern subspecies (*queenslandica* and *crassifolia*) and putative southern subspecies (*moluccana* and *pedicellata*) (Figure 3). Furthermore, the petiole length separated the seedlings of putative subspecies *moluccana* and *pedicellata* but did not separate seedlings of *queenslandica* and *crassifolia*, both of which had the largest leaves (due to leaf width), whereas the southern putative subspecies *moluccana* and *pedicellata* seemed to have narrower leaves (Figure 3). Interestingly, a geographic pattern was noted in which samples from QLD (putative subspecies *queenslandica* and *crassifolia*) grouped together on one side of the plot and samples from NSW (putative subspecies *moluccana* and *pedicellata*) on the other side (Figure 3). The petiole length and leaf ratio (length:width) contributed most to explain these differences among subspecies. The diameter and length of the primary seedling stem were also informative, providing functional traits revealed under common garden conditions. The first two canonical discriminant functions explained 99% of the morphological variation between the four subspecies, with Wilks’ lambda test of functions being significant (Wilks’ lambda = 0.033; *p* < .0001). From the 253 samples, 113 were misidentified by the analysis, mostly due to incorrect assignments to putative subspecies *queenslandica* and *crassifolia*, suggesting that there is no clear morphological differentiation among these two putative subspecies at young stages. At 18 months, there was an even greater overlap among subspecies (Figure S1).

### 3.3 Chloroplast haplotype network

**FIGURE 4:**
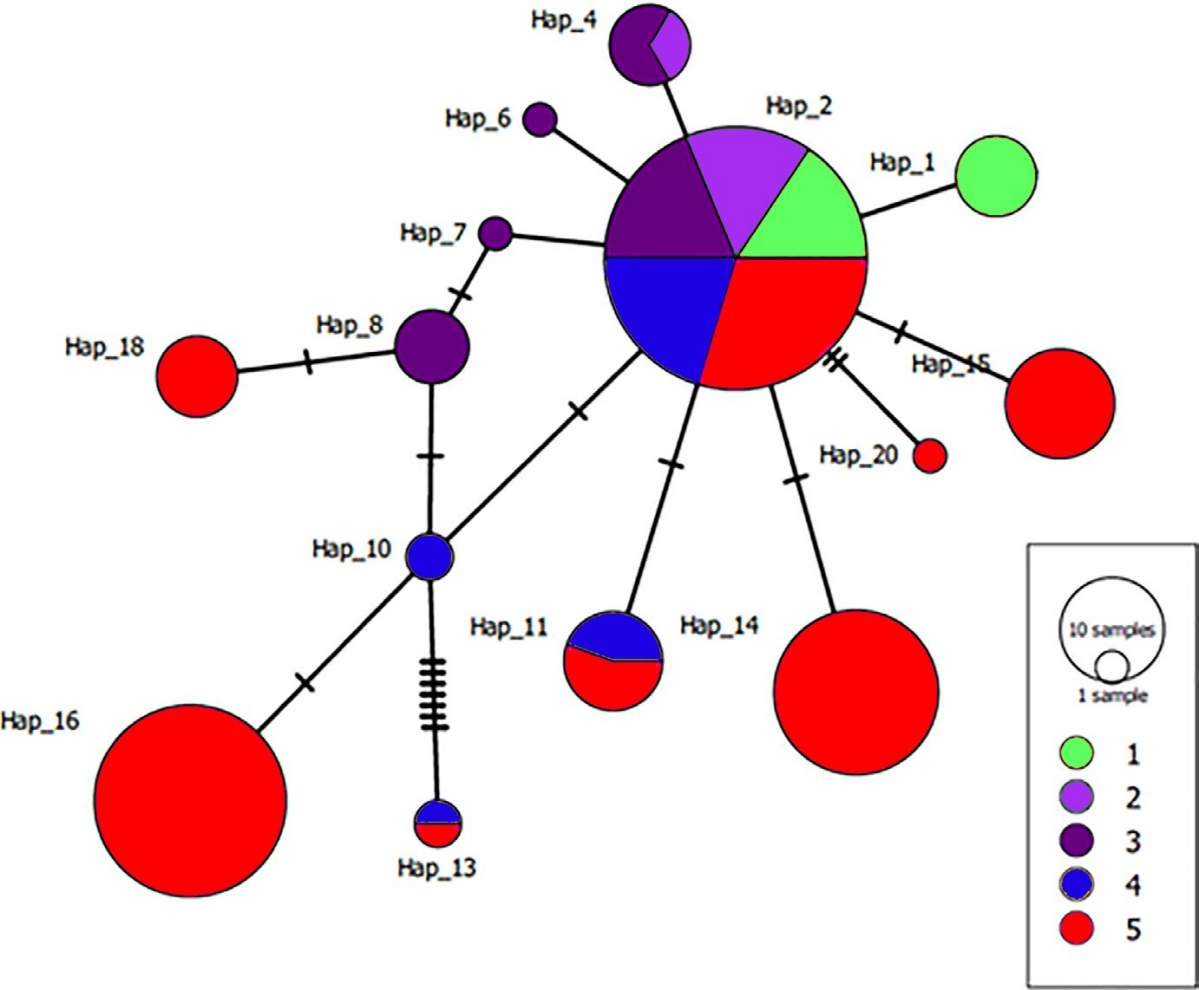
Haplotype network constructed with the chloroplast intergenic region *psbA‐trnH* (*n* = 169). Five regions (as per Figure 2) encompassing the four putative subspecies, from north to south, *crassifolia* (Region 1 = green), *queenslandica* (Region 2 = light purple; Region 3 = dark purple), *pedicellata* (Region 4 = blue), and *moluccana* (Region 5 = red)

Haplotype network analysis of the chloroplast intergenic region *psbA‐trnH* revealed twenty haplotypes with a main haplotype in a central position shared by individuals belonging to the five regions and the four subspecies (Figure 4). Only fourteen haplotypes are visible because PopArt ignores indels in the data. Although this ancestral polymorphism was found to be shared across the five regions, each region also had unique haplotypes, suggesting geographic structuring and a recent expansion and differentiation. Haplotype 4 was shared between samples of Regions 2 and 3 (Crediton State Forest and Calliope in QLD – putative subspecies *queenslandica*). Similarly, Haplotypes 11 and 13 were shared between samples of Regions 4 and 5 (putative subspecies *pedicellata* and *moluccana*). Haplotype 11 included individuals from northern NSW (Bom State Forest) and from the Sydney Basin (Bellevue Park), whereas Haplotype 13 included samples from near the Hunter Valley (Wallaroo) and the Sydney Basin (Scheyville National Park) (Figure 4).

### 3.4 Genetic structure and differentiation

**FIGURE 5:**
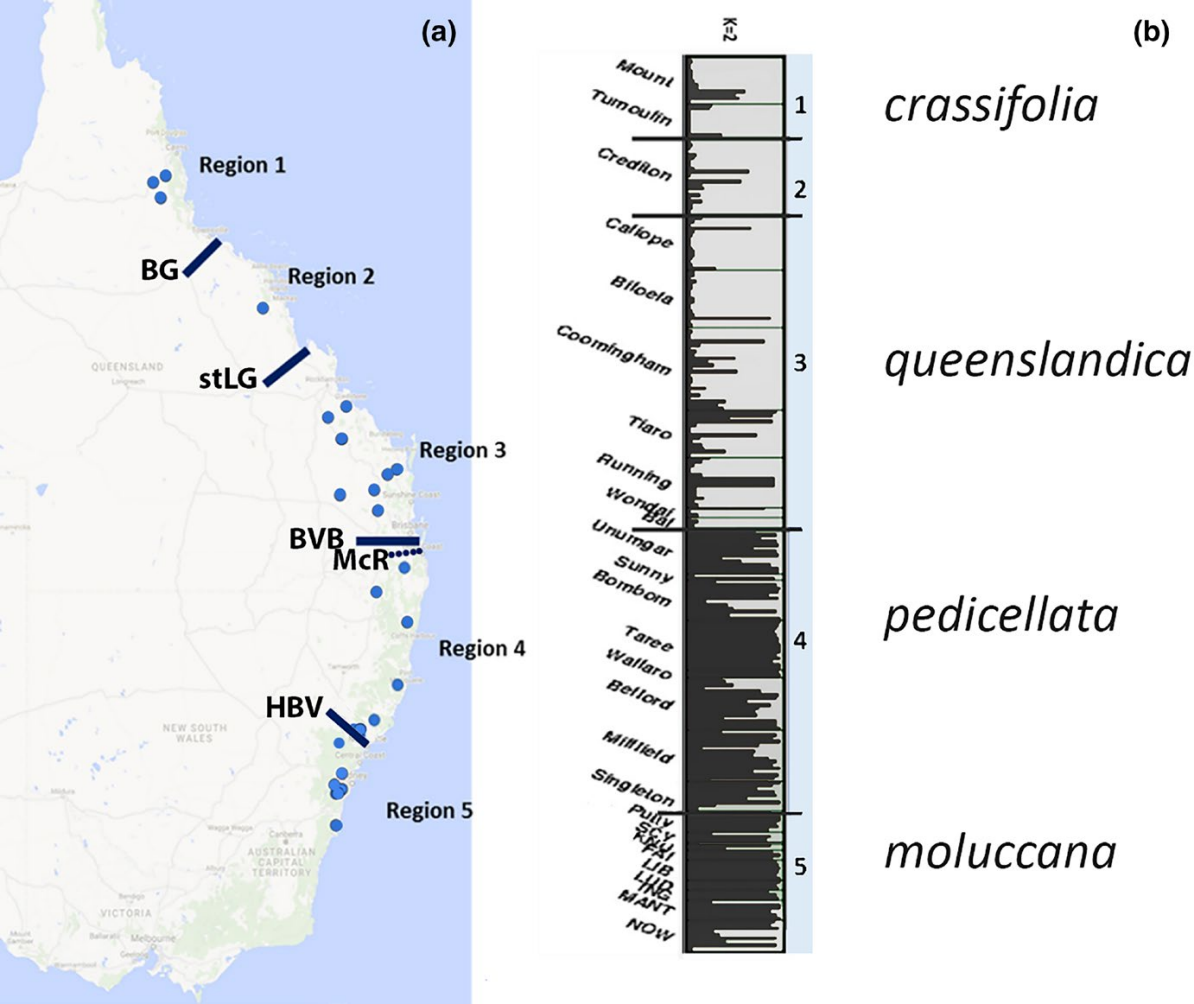
(a) Populations collected from five regions delimited by four recognized biogeographic barriers in eastern Australia (Bryant & Krosch, 2016; Chapple et al., 2011). (b) Genetic structure of *Eucalyptus moluccana*. K = 2 identified two clusters, with populations sorted by latitude, from north to south. Each bar represents an individual plant, and grey and black indicate cluster assignments. Biogeographic barriers are separated by heavy black lines. Locations of populations are listed on the left, and Regions 1 to 5 are listed on the right. Biogeographic barriers are marked as Burdekin Gap (BG), and St Lawrence Gap (StLG), Brisbane Valley Barrier (BVB)/McPherson Range (McR), and Hunter Valley (HVB). According to Gillison (1976) *E. moluccana* subspecies *crassifolia* is present in Region 1, subspecies *queenslandica* is present in Regions 2 and 3, subspecies *pedicellata* is mainly present in Region 4, and subspecies *moluccana* is present mainly in Region 5

The optimal cluster numbers in the STRUCTURE analysis were determined using the ∆K method developed by Evanno et al. (2005), which provided the optimal value of K = 2 (Figure 5b, Table S3, Figure S2). Admixture was detected in all clusters. According to the clustering of K = 2, a higher proportion of green (Figure 5b) was seen in samples from the northern latitudes (QLD) corresponding mainly to the regions that contained the putative subspecies *crassifolia* and *queenslandica*; and a second cluster with a higher proportion of red represented by samples mostly from southern populations (NSW) corresponding to regions that contained the putative subspecies *pedicellata* and *moluccana*. The structure was detected between the QLD and NSW populations separated by the BVB biogeographic barrier. Notably, the second best number of clusters estimated by the Evanno method was K = 5 (Figure S3). The clustering of mainly three groups in QLD was consistent with the BG and StLG barriers (Chapple et al., 2011; Cracraft, 1991; Ford, 1987). Although there was no sharp differentiation across these bioregions (presumably due to extensive gene flow), some patterns were detected. The clustering of K = 5 seemed to coincide with the five predicted regions split by four barriers; however, the substructuring analysis suggested that the St Lawrence Gap does not act as a geographic barrier in this species (Figure S4). Exemptions to this trend were some samples from Biloela, located in Region 3, which clustered with samples of Region 2 – Crediton State Forest. The second example were samples from Bom State Forest located in Region 4 in NSW which clustered with samples from Region 5 – Sydney Basin. Highly admixed populations were found in Belford (Hunter Valley) and Nowra (south of Sydney) challenging the clustering. The northernmost populations (Region 1) were clearly isolated from the rest; the three analyzed populations from this region belonged to the putative subspecies *crassifolia*. The putative subspecies *queenslandica* was mainly represented by two clusters (samples with high proportions of blue and yellow). The putative subspecies *pedicellata* was represented by one cluster with high admixture (mainly samples with a high proportion of red). Finally, the putative subspecies *moluccana* was represented by one cluster with individuals that had a high proportion in orange. Further substructuring (following approaches used by Janes et al., 2017) of the two clusters (QLD and NSW populations) resulted in samples of QLD populations to have an optimal substructuring cluster number of 2, whereas samples of NSW populations had a substructuring cluster number of 3 (Figure S4).

**FIGURE 6:**
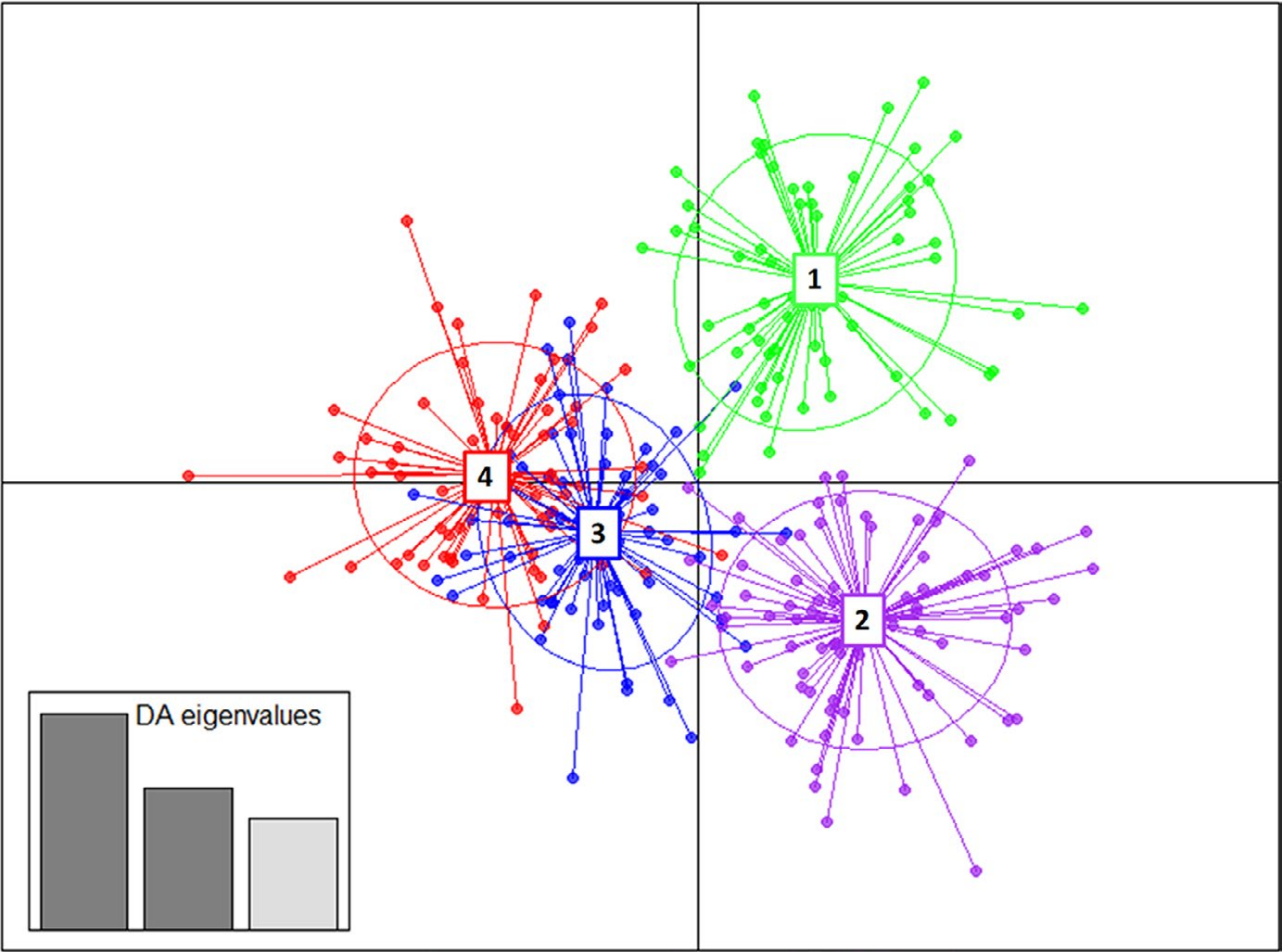
Discriminant analysis of principal components (DAPC) detected four genetic groups: Cluster 1 (green) contained all of the samples from the subspecies *crassifolia* (Region 1) and was more distant from the other clusters. Cluster 2 (purple) was predominantly formed by individuals from Region 2, hypothesized to be subspecies *queenslandica*. Cluster 3 (blue) was formed by individuals from Region 3. Cluster 4 (red) was formed by individuals of Regions 4 and 5 (subspecies *pedicellata* and *moluccana*)

DAPC detected four genetic groups: Cluster 1 contained all the samples from the subspecies *crassifolia* (Region 1) and was more distant from the other clusters. Cluster 2 was predominantly formed by individuals from Regions 2 and some of 3, hypothesized to be subspecies *queenslandica*, and a low proportion of the other subspecies. Cluster 3 was formed mainly by individuals from Region 3. Cluster 4 was formed mainly by individuals of Regions 4 and 5 (subspecies *pedicellata* and *moluccana*) (Figure 6). Clusters 3 and 4 overlapped extensively and corresponded to subspecies *pedicellata* and *moluccana*. Based on the results from STRUCTURE, we defined two genetic groups that corresponded to QLD and NSW. From K = 3 to 10, the STRUCTURE analysis (Figure S3) showed that subspecies *crassifolia* exhibiting less admixture. Global AMOVA and Pairwise FST comparisons were calculated for either (a) Regions 1 to 5 and (b) QLD and NSW populations following results represented in Figure 5 (Tables 2 and 3). Consistent with STRUCTURE, the highest FST on pairwise difference was detected for Region 1 (i.e., subspecies *crassifolia* was most distant). Although the PCoA analysis (Figure S5) showed some samples from the same region tended to be more closely related (e.g., samples from Region 1 or subspecies *crassifolia*), there was a high overlap between all regions. The Mantel test revealed that there was a significant correlation between the geographic and genetic distance (Rxy = 0.25, *p* = .001).

**TABLE 2:** Global AMOVA results as a weighted average over ten microsatellite loci comparing all five regions with two broad regions (QLD and NSW) across the entire distribution for *Eucalyptus moluccana*

Source	*df*	SS	Est. Var.	% among 5 regions	*df*	SS	Est. Var.	% between NSW and QLD
Among regions	4	72.166	0.144	4%	1	28.234	0.091	2%
Within regions	531	2061.407	3.882	96%	534	2,105.339	3.943	98%
Total	535	2,133.573	4.026	100%	535	2,133.573	4.033	100%

**TABLE 3:** Population pairwise FSTs among the five geographic regions

Region1	Region2	Region3	Region4	Region5	
0.000					Region1
0.062	0.000				Region2
0.048	0.037	0.000			Region3
0.059	0.051	0.023	0.000		Region4
0.075	0.065	0.031	0.015	0.000	Region 5

Note

Distance method: pairwise differences by regions (clusters). Cluster number matches the regions in Figure 2a. According to Gillison (1976), Region 1 has individuals of putative subspecies *crassifolia*, Regions 2 and 3 have mainly individuals of putative subspecies *queenslandica*, Region 4 has mainly individuals from putative subspecies *pedicellata*, and Region 5 has mainly individuals from putative subspecies *moluccana*.

## 4 DISCUSSION

We demonstrate clear population structuring in the common and widespread *E. moluccana* that matches some biogeographic barriers described for eastern Australia (Bryant & Krosch, 2016; Chapple et al., 2011) and provide the first genetic evidence for some of the subspecies proposed by Gillison (1976), with *E. moluccana* subspecies *crassifolia* being the most differentiated. Higher genetic differentiation between QLD and NSW populations was recovered, associated with the split geographically by the McPherson Range barrier. Weak differentiation into a total of five genetic clusters was evident (pairwise ranged from FST of 0.015 to 0.075) compared with other species of *Eucalyptus* (e.g., Smith et al., 2003). A similar pattern has been found in *E. grandis* for which genetic diversity was split into five regions with weak differentiation (Jones et al., 2006). Interestingly, *E. grandis* is a species with a stronger affinity to mesic environments and has less occupation of regions across central Queensland than *E. moluccana* which occurs in dry woodlands and has a wider distribution. Although isolation by distance might play a role in the genetic structure of *E. moluccana*, the clustering analyses support the hypothesis that geographic barriers have played a role in the evolution of this species. Moreover, the recent expansion detected in our chloroplast network analysis is consistent with previous work in the genus *Eucalyptus*, particularly in the subgenus Symphyomyrtus in which an explosive radiation has occurred (Thornhill et al., 2019). This may have resulted in the issue that morphometric analyses of *E. moluccana* seedlings did not unequivocally succeed in separating the putative subspecies, yet similar approaches were successful in separating species of the group of eastern grey boxes (Flores‐Rentería et al., 2017).

### 4.1 Impact of individual geographic barriers on *E. moluccana* populations

The Burdekin Gap (BG) contains dry woodlands and lowland savanna that separate the northern wet tropics rainforests and monsoonal habitats from the southern mesic habitats. Populations of *E. moluccana* (Cluster 1) to the north of the BG were the most differentiated ones. A similar pattern was found in *E. grandis* and *C. citriodora* (Jones et al., 2006; Shepherd et al., 2008). Interestingly, the northern *E. moluccana* populations occur at a higher elevation than all other populations. This region is more inland, in the rain shadow of the GDR, and substantially drier than the coastal region with rainforests. The *E. moluccana* populations north to the BG are isolated and clustered morphologically and genetically as subspecies *crassifolia*. Other taxa that have experienced structuring due to BG included freshwater fish species and other water‐dependent land animals, such as the delicate skink (Chapple et al., 2011). Within the lowland savannah of the BG, no *E. moluccana* has been reported except for individuals on Mount Abbott National Park.

The St Lawrence Gap (StLG) between Mackay and Rockhampton in central QLD contains open woodlands and savannah (Bryant & Krosch, 2016). Grey box has a low representation within this region, with some individuals reported in the Eugene State Forest and surrounding areas. This gap has a drier and warmer climate than adjacent uplands (Webb & Tracey, 1981). Although this barrier matches the break for individuals in genetic Cluster 3 of the structure analysis (mostly purple, Figure 5), the subpopulation in Biloela shared similar patterns as Region 2 (mostly lilac, Figure 5). The lack of a clear differentiation between Regions 2 and 3 was detected in the substructuring analysis (Figure S4) and the DAPC. Therefore, this barrier might not have a strong isolating effect for Clusters 2 and 3. However, it is an important barrier to species of skinks, birds, pademelon, insects, and orchids occupying wetter environments (Bryant & Krosch, 2016). More relevant is the lack of *E. moluccana* in the region north of the StLG, which corresponds to a different bioregion (Brigalow Belt) and with a different vegetation community that may act as a barrier for *E. moluccana*.

The McPherson Range (McR) is a mountain block of rainforest along the border of QLD and NSW. The structure analysis (K = 2) supports McR acting as a barrier to gene flow between the putative northern and southern subspecies of *E. moluccana*. This barrier is a hybrid zone for several bird species and a barrier for lowland and dry forest plant species (Chapple et al., 2011; Crisp et al., 1995). It splits QLD and NSW populations of invertebrates (McLean et al., 2008), for example, crayfish *Euastacus* sp. (Ponniah & Hughes, 2004), vertebrates (James & Moritz, 2000), and plants (Milner et al., 2012). The McR limits distribution of snails, assassin spiders, and some plants such as *Ceratopetalum apetalum* (Bryant & Krosch, 2016). *Eucalyptus moluccana* grows in the Brisbane Valley north of the McR, at lower density and more scattered, and some stands are restricted to higher altitudes on Flinders Peak Conservation Park, Moogerah Peaks National Park, and other restricted areas; therefore, BVB might also reduce gene flow.

The Hunter Valley (HVB) is a dry habitat and acts as a biological barrier (Chapple et al., 2011; Milner et al., 2012) to many animal (Chapple et al., 2011; Donnellan et al., 1999; Joseph et al., 2008) and plant species of mesic environments (Playford et al., 1993; Di Virgilio et al., 2012; Heslewood et al., 2014; Milner et al., 2012, but see Shepherd et al., 2012). It is also potentially one of the oldest biogeographic barriers of eastern Australia (Milner et al., 2012). However, the dry sclerophyllous *E. moluccana* grows abundantly throughout this area, including also populations to the west of the HVB. Therefore, it is unlikely a barrier to grey box, but rather the more mesic vegetation (e.g., *C. apetalum*) on the ranges to the south may reduce gene flow between the Hunter Valley and the Sydney Basin. Furthermore, populations of *E. moluccana* in the Sydney region are surrounded by an elevated sandstone belt formed by the Blue Mountains and the Hornsby Plateau on which *E. moluccana* does poorly, and these formations may form a barrier against populations further north. Moreover, the population from Putty close to the Hunter Valley, but part of the Hawkesbury–Nepean catchment of the Sydney Basin, clustered with samples of the Sydney Region, an indication that river systems may influence the structure of this species.

Interestingly, samples from the Bom State Forest of northern NSW clustered with samples of the Sydney Basin more than 600 km apart. Current revegetation programs use predominantly local seeds (Mortlock, 2000); we did not find records of past reforestation efforts, yet in the past, the source of some planted forest stands might be from more distant populations, and this might confound the analysis. Although no strong evidence was found to support the distinction between subspecies *pedicellata* and *moluccana*, most of the genetic structure patterns seem consistent with the geographic barriers and with the subspecies described by Gillison (1976), suggesting that revegetation efforts have not dramatically affected the natural patterns.

### 4.2 Subspecies differences and the geographic barriers

Gillison (1976) proposed four subspecies of *E. moluccana*, despite most of the morphological features varying clinally. This clinal pattern was consistent with the levels of admixture found in our population genetic analyses. Notably, putative subspecies *crassifolia* was separated from the other three subspecies based on its extremely broad, almost completely orbicular, thickened leaves with undulate margins of mature individuals (Gillison, 1976). Gillison (1976) also noted for this subspecies that the flower number per peduncle is commonly 11 to 15, and the box‐type bark proportion can be as high as 80%, often up to or beyond the primary branches (e.g., at Scrubby Creek near Ravenshoe in the Atherton Tablelands). He found a smaller leaved, distinctly urceolate form west toward the drier country near Innot Hot Springs and Mount Garnet, where the tree size was also reduced. These northern populations, belonging to the subspecies *crassifolia*, are disjunct from all other populations of *E. moluccana* further south. Pryor and Johnson (1971) have tentatively elevated these northern populations to their own species rank, but Gillison (1976) assigned them to the subspecies rank *crassifolia*. However, he noticed a very distinctive set of features in comparison to the other three subspecies, for example, individuals from subspecies *crassifolia* have rudimentary lignotubers and are associated with more mesic coastal communities than the prominent lignotubers found in the other subspecies (Gillison, 1976). Although our morphological analysis of seedling leaves did not separate subspecies *crassifolia* in its own cluster, the genetic analysis suggests genetic differentiation of subspecies *crassifolia* from the other subspecies. More genetic and ecological studies are needed to confirm its rank as subspecies and should consider potential interspecific hybridization that has been documented extensively in box eucalypts (Flores‐Rentería et al., 2017).

The analysis of juvenile leaf morphometrics showed that subspecies *crassifolia* and subspecies *queenslandica* were more similar in appearance and partially overlapped with subspecies *moluccana*, whereas subspecies *pedicellata* was the most distinctive. Consistent with observations by Gillison (1976), the vector separating the QLD subspecies (*crassifolia* and *queenslandica*) from the NSW subspecies (*pedicellata* and *moluccana*) was the leaf ratio and one of the vectors further separating subspecies *pedicellata* from the other species was the pedicel length at 6 months of age. However, despite the small sample size of subspecies *pedicellata*, it had the highest variance suggesting inclusion of more samples could reduce this separation. The lack of genetic and morphological differentiation between subspecies *moluccana* and subspecies *pedicellata* suggest they are likely just one subspecies. At 18 months, the slight differences in morphology decreased, and this is expected as leaves of *Eucalyptus* are heteroblastic, and they seem to exhibit significant differences during seedling stage but change during the development to be more uniform at maturity. Thus, leaf morphology of seedlings does not seem to be as effective to differentiate among subspecies as it has been found when comparing different species (Bean, 2009; Ladiges et al., 1981; Rutherford et al., 2017), including between the group of the eastern grey box eucalypt species (Flores‐Rentería et al., 2017). According to Gillison (1976), the most morphologically diverse communities of *E. moluccana* are found in the coastal regions of northern and central NSW. This suggests that *E. moluccana* may have radiated from this region. However, our chloroplast haplotype network shows a central haplotype with shared haplotypes among the four subspecies; therefore, detection of ancestral populations was less evident.

## 5 CONCLUSIONS

Using genetic and morphological data, we have revealed that geographic and biological barriers along eastern Australia have shaped the population genetic structure of *E. moluccana* and contributed to the subspeciation process. Despite the widespread admixture, our analyses separated northern (QLD) from southern (NSW) populations which are divided by the BVB/McR. Additionally, the subspecies *crassifolia* which was more genetically distant is also more isolated. Admixture among the subspecies was readily detected, suggesting recent divergence or contact. Our study provides the first in‐depth genetic characterization of *E. moluccana*, a widely distributed and ecologically important tree species that is dominant in grassy woodlands of eastern Australia. Its population genetic diversity and structure are important in the context of conservation and revegetation efforts of grassy woodlands as highlighted for a close relative, the inland grey box *Eucalyptus microcarpa* (Jordan et al., 2016). Across its range, many *E. moluccana* stands have been cleared for agriculture, and in regions such as the Sydney Basin also for the development of housing and industrial estates. Given that its subspecies structure between regions is separated by major biogeographic barriers, it may be important to not mix sources across regions for revegetation purposes. Conversely, this species is distributed across a large climatic gradient, with potential for local climatic adaptation. This could provide opportunities for revegetation efforts to source provenances from regions that better represent future climatic conditions as predicted in the context of climate change. This might be particularly important to investigate further for populations of *E. moluccana* that have suffered from substantial defoliation due to a climate‐driven outbreak of a host‐specific psyllid species (Hall et al., 2015, 2019).

## CONFLICT OF INTEREST

None declared.

## AUTHOR CONTRIBUTIONS

**Lluvia Flores‐Rentería:** Data curation (lead); Formal analysis (lead); Investigation (equal); Software (lead); Validation (lead); Visualization (lead); Writing‐original draft (lead); Writing‐review & editing (lead). **Paul D. Rymer:** Conceptualization (supporting); Data curation (supporting); Funding acquisition (supporting); Project administration (supporting); Resources (supporting); Supervision (supporting); Validation (supporting); Writing‐review & editing (supporting). **Niveditha Ramadoss:** Formal analysis (supporting); Software (supporting); Visualization (supporting); Writing‐original draft (supporting); Writing‐review & editing (supporting). **Markus Riegler:** Conceptualization (lead); Data curation (supporting); Funding acquisition (lead); Investigation (equal); Project administration (lead); Resources (supporting); Supervision (lead); Validation (supporting); Writing‐review & editing (supporting).

## Supporting information

Supplementary Material

## Data Availability

All data used in this article are deposited in Dryad and are available via the following link https://datadryad.org/stash/share/C‐4HdHrE8KgrGGYXK‐NYZEOn2uBPuMq2xIQFF8ek2iQ.
