# Supplementary material for: Major biogeographic barriers in eastern Australia have shaped the population structure of widely distributed Eucalyptus moluccana and its putative subspecies
Source: Ecol Evol. 2021 Sep 30;11(21):14828–42. doi: 10.1002/ece3.8169 (PMC8571587; doi:10.1002/ece3.8169)
Supplement: Supplementary file 1 — Supplementary Material [file ECE3-11-14828-s001.docx]

**Major biogeographic barriers in eastern Australia have shaped the population structure of widely distributed *Eucalyptus moluccana* and its putative subspecies**

Lluvia Flores-Rentería^a^; Paul D. Rymer^b^; Niveditha Ramadoss^a^; Markus Riegler^b^

^a^ Department of Biology, San Diego State University, San Diego, CA 92182, USA

^b^ Hawkesbury Institute for the Environment, Western Sydney University, Locked Bag 1797, Penrith NSW 2751, Australia

**Supplementary Material**

**Table S1.** Microsatellite primer sequences and expected size range in species for which they were developed.

| **Accession** | **Forward** | **Reverse** | **Repeat motif** | **Size range** | **Target species** |
| --- | --- | --- | --- | --- | --- |
| BV682066 | TGTTCCTGGATTGTCACTTA | ATCCAGATTGAGCACAGAC | (CT)20 | 105 | *E. grandis* |
| BV682112 | AAGAACATGCAGCGGAGA | TTGTGATGGACCACTCAATG | (CT)2C(CT)23 | 129 | *E. grandis* |
| BV682167 | GTCATTGTTGCGATCACTGC | ACGTGACTTGGTTGATCTGC | (CT)21 | 222 | *E. grandis* |
| *El14* | ACCTTAGAAAAGTCGAAGCATC | ACCTCCACATACCAGTCAC | (GT)13(AG)15 | 184 | *E. leucoxylon* |
| *El27* | GTCTTTCAGAGAGCGATTTC | ATTTCTGTGATTTGCTTTGG | (TGG)3(AGC)12 | 307 | *E. leucoxylon* |
| EU694398 |  |  | (CT)16 | 90-129 | *E. sieberi* |
| EU699745 | AAAACGAACCACCCTTCCTC | CCTTTTGATGGGACTTGGTG |  |  | *E. globulus* |
| EU699755 | CCAGGGAAAACAATTCAAGC | GAGCGACAAACCCAAGTTTC | (GAA)5(GAG)8(GAA)5 | 266-302 | *E. globulus* |
| GF101862 | GTCGAGTTGAGTTCGCTTCC | AGTGAATCGGGAGAGGAGGT | (CTCCTG)26 | 287-299 | *E. grandis* |
| GF101866 | GGAGGAGGAGGAACAGGAAC | GCACCCGGTTCTTAAATCAA | (CCCT)21 | 203-219 | *E. grandis* |

**Table S2.** Sequences of universal primers and their associated fluorescence labels.

| **Tail** | **Label** | **Sequence 5’- 3’** | **Tm (°C)** | **References** |
| --- | --- | --- | --- | --- |
| M13 | FAM | TGTAAAACGACGGCCAGTG | 60 | Schuelke, 2000 |
| dys | VIC | GACTATGTGCGTGAGTGCAGG | 61 | Missiaggia and Grattapaglia, 2006 |
| d12 | PET | ACCAACCTAGGAAACACAGGC | 60 | Missiaggia and Grattapaglia, 2006 |
| TailC | NED | CAGGACCAGGCTACCGTG | 60 | Blacket *et al*., 2012 |

Table S3. Inferred ancestry of individuals from STRUCTURE analysis in K=2.

| Label (%Miss) :  Inferred clusters | | | |
| --- | --- | --- | --- |
| 1 | MGAR1 (0): | 0.026 | 0.974 |
| 2 | MGAR2 (0): | 0.056 | 0.944 |
| 3 | MGAR3 (0): | 0.024 | 0.976 |
| 4 | MGAR4 (0): | 0.022 | 0.978 |
| 5 | MGAR5 (0): | 0.059 | 0.941 |
| 6 | MGAR6(10): | 0.070 | 0.93 |
| 7 | MGAR7 (0): | 0.042 | 0.958 |
| 8 | MGAR8 (5): | 0.087 | 0.913 |
| 9 | MGAR9 (0): | 0.128 | 0.872 |
| 10 | MGAR10 (0): | 0.103 | 0.897 |
| 11 | MGAR11 (5): | 0.721 | 0.279 |
| 12 | MGAR12 (0): | 0.608 | 0.392 |
| 13 | MGAR13 (0): | 0.617 | 0.383 |
| 14 | MGAR14 (0): | 0.029 | 0.971 |
| 15 | TUM1 (0): | 0.240 | 0.76 |
| 16 | TUM2 (5): | 0.468 | 0.532 |
| 17 | TUM3 (5): | 0.028 | 0.972 |
| 18 | TUM4 (0): | 0.017 | 0.983 |
| 19 | TUM5 (0): | 0.032 | 0.968 |
| 20 | TUM6 (0): | 0.019 | 0.981 |
| 21 | TUM7 (0): | 0.022 | 0.978 |
| 22 | TUM8 (0): | 0.030 | 0.97 |
| 23 | TUM9 (0): | 0.023 | 0.977 |
| 24 | TUM10 (0): | 0.431 | 0.569 |
| 25 | CRE1 (0): | 0.130 | 0.87 |
| 26 | CRE2 (0): | 0.019 | 0.981 |
| 27 | CRE3 (0): | 0.041 | 0.959 |
| 28 | CRE4(10): | 0.023 | 0.977 |
| 29 | CRE5 (0): | 0.013 | 0.987 |
| 30 | CRE6 (0): | 0.076 | 0.924 |
| 31 | CRE7 (0): | 0.086 | 0.914 |
| 32 | CRE8 (0): | 0.020 | 0.98 |
| 33 | CRE9 (0): | 0.028 | 0.972 |
| 34 | CRE10 (0): | 0.073 | 0.927 |
| 35 | CRE11(10): | 0.696 | 0.304 |
| 36 | CRE12 (0): | 0.033 | 0.967 |
| 37 | CRE13 (0): | 0.014 | 0.986 |
| 38 | CRE14 (0): | 0.645 | 0.355 |
| 39 | CRE15 (0): | 0.170 | 0.83 |
| 40 | CRE16 (0): | 0.136 | 0.864 |
| 41 | CRE17 (0): | 0.014 | 0.986 |
| 42 | CRE18 (0): | 0.099 | 0.901 |
| 43 | CRE19 (0): | 0.020 | 0.98 |
| 44 | CRE20 (0): | 0.170 | 0.83 |
| 45 | CRE21 (0): | 0.055 | 0.945 |
| 46 | CRE22 (0): | 0.037 | 0.963 |
| 47 | CRE23 (0): | 0.019 | 0.981 |
| 48 | CAL1 (0): | 0.106 | 0.894 |
| 49 | CAL2 (0): | 0.129 | 0.871 |
| 50 | CAL3 (0): | 0.059 | 0.941 |
| 51 | CAL4 (0): | 0.027 | 0.973 |
| 52 | CAL5 (0): | 0.573 | 0.427 |
| 53 | CAL6 (0): | 0.046 | 0.954 |
| 54 | CAL7 (0): | 0.041 | 0.959 |
| 55 | CAL8 (0): | 0.017 | 0.983 |
| 56 | CAL9 (0): | 0.026 | 0.974 |
| 57 | CAL10 (0): | 0.017 | 0.983 |
| 58 | CAL11 (0): | 0.050 | 0.95 |
| 59 | CAL12 (0): | 0.050 | 0.95 |
| 60 | CAL13 (0): | 0.052 | 0.948 |
| 61 | CAL14 (0): | 0.056 | 0.944 |
| 62 | CAL15 (0): | 0.018 | 0.982 |
| 63 | CAL16 (0): | 0.049 | 0.951 |
| 64 | CAL17 (0): | 0.293 | 0.707 |
| 65 | BIOL1 (0): | 0.057 | 0.943 |
| 66 | BIOL2 (0): | 0.055 | 0.945 |
| 67 | BIOL3 (0): | 0.030 | 0.97 |
| 68 | BIOL4 (0): | 0.083 | 0.917 |
| 69 | BIOL5 (0): | 0.036 | 0.964 |
| 70 | BIOL6 (0): | 0.043 | 0.957 |
| 71 | BIOL7 (0): | 0.027 | 0.973 |
| 72 | BIOL8 (0): | 0.030 | 0.97 |
| 73 | BIOL9 (5): | 0.052 | 0.948 |
| 74 | BIOL10 (0): | 0.077 | 0.923 |
| 75 | BIOL11 (0): | 0.037 | 0.963 |
| 76 | BIOL12 (0): | 0.019 | 0.981 |
| 77 | BIOL13 (0): | 0.063 | 0.937 |
| 78 | BIOL14 (0): | 0.050 | 0.95 |
| 79 | BIOL15 (0): | 0.858 | 0.142 |
| 80 | BIOL16 (0): | 0.034 | 0.966 |
| 81 | BIOL17 (0): | 0.048 | 0.952 |
| 82 | COOM1 (0): | 0.015 | 0.985 |
| 83 | COOM2 (0): | 0.034 | 0.966 |
| 84 | COOM3 (0): | 0.026 | 0.974 |
| 85 | COOM4 (0): | 0.036 | 0.964 |
| 86 | COOM5 (0): | 0.825 | 0.175 |
| 87 | COOM6 (0): | 0.121 | 0.879 |
| 88 | COOM7 (0): | 0.033 | 0.967 |
| 89 | COOM8 (0): | 0.098 | 0.902 |
| 90 | COOM9(10): | 0.089 | 0.911 |
| 91 | COOM10 (0): | 0.526 | 0.474 |
| 92 | COOM11 (5): | 0.237 | 0.763 |
| 93 | COOM12 (0): | 0.237 | 0.763 |
| 94 | COOM13 (0): | 0.049 | 0.951 |
| 95 | COOM14 (0): | 0.511 | 0.489 |
| 96 | COOM15 (0): | 0.026 | 0.974 |
| 97 | COOM16 (0): | 0.050 | 0.95 |
| 98 | COOM17 (0): | 0.048 | 0.952 |
| 99 | COOM18 (0): | 0.021 | 0.979 |
| 100 | COOM19 (0): | 0.194 | 0.806 |
| 101 | COOM20 (0): | 0.035 | 0.965 |
| 102 | COOM21 (5): | 0.103 | 0.897 |
| 103 | COOM22 (0): | 0.027 | 0.973 |
| 104 | COOM23 (0): | 0.368 | 0.632 |
| 105 | COOM24 (0): | 0.298 | 0.702 |
| 106 | COOM25 (0): | 0.379 | 0.621 |
| 107 | TIA1 (0): | 0.953 | 0.047 |
| 108 | TIA2 (0): | 0.924 | 0.076 |
| 109 | TIA3 (0): | 0.676 | 0.324 |
| 110 | TIA4 (0): | 0.925 | 0.075 |
| 111 | TIA5(10): | 0.129 | 0.871 |
| 112 | TIA6 (5): | 0.037 | 0.963 |
| 113 | TIA7 (0): | 0.025 | 0.975 |
| 114 | TIA8 (0): | 0.720 | 0.28 |
| 115 | TIA9 (0): | 0.112 | 0.888 |
| 116 | TIA10 (0): | 0.045 | 0.955 |
| 117 | TIA11 (0): | 0.192 | 0.808 |
| 118 | TIA12 (0): | 0.203 | 0.797 |
| 119 | TIA13 (0): | 0.405 | 0.595 |
| 120 | TIA14 (0): | 0.019 | 0.981 |
| 121 | RUNC1 (0): | 0.217 | 0.783 |
| 122 | RUNC2 (0): | 0.138 | 0.862 |
| 123 | RUNC3 (0): | 0.060 | 0.94 |
| 124 | RUNC4 (0): | 0.034 | 0.966 |
| 125 | RUNC5 (0): | 0.032 | 0.968 |
| 126 | RUNC6 (0): | 0.198 | 0.802 |
| 127 | RUNC7 (0): | 0.928 | 0.072 |
| 128 | RUNC8 (0): | 0.924 | 0.076 |
| 129 | RUNC9 (0): | 0.939 | 0.061 |
| 130 | RUNC10 (0): | 0.103 | 0.897 |
| 131 | RUNC11 (0): | 0.113 | 0.887 |
| 132 | RUNC12 (0): | 0.082 | 0.918 |
| 133 | RUNC13(10): | 0.263 | 0.737 |
| 134 | RUNC14 (0): | 0.059 | 0.941 |
| 135 | RUNC15 (5): | 0.079 | 0.921 |
| 136 | WON1 (0): | 0.826 | 0.174 |
| 137 | WON2 (0): | 0.016 | 0.984 |
| 138 | WON3 (0): | 0.102 | 0.898 |
| 139 | BAL1 (0): | 0.017 | 0.983 |
| 140 | BAL2 (0): | 0.115 | 0.885 |
| 141 | BAL3 (0): | 0.062 | 0.938 |
| 142 | UNU10 (0): | 0.459 | 0.541 |
| 143 | UNU12 (0): | 0.700 | 0.3 |
| 144 | UNU13 (0): | 0.946 | 0.054 |
| 145 | UNU14 (0): | 0.773 | 0.227 |
| 146 | UNU15 (0): | 0.941 | 0.059 |
| 147 | UNU16(10): | 0.964 | 0.036 |
| 148 | UNU17 (5): | 0.775 | 0.225 |
| 149 | UNU2 (0): | 0.963 | 0.037 |
| 150 | UNU3 (0): | 0.972 | 0.028 |
| 151 | UNU4 (0): | 0.305 | 0.695 |
| 152 | UNU5 (0): | 0.574 | 0.426 |
| 153 | UNU6 (0): | 0.894 | 0.106 |
| 154 | UNU7(10): | 0.977 | 0.023 |
| 155 | UNU8(10): | 0.976 | 0.024 |
| 156 | SUN1 (0): | 0.072 | 0.928 |
| 157 | SUN2(10): | 0.947 | 0.053 |
| 158 | BOM10 (0): | 0.274 | 0.726 |
| 159 | BOM11 (0): | 0.951 | 0.049 |
| 160 | BOM12 (0): | 0.984 | 0.016 |
| 161 | BOM13 (5): | 0.957 | 0.043 |
| 162 | BOM14 (0): | 0.859 | 0.141 |
| 163 | BOM15 (0): | 0.968 | 0.032 |
| 164 | BOM2 (0): | 0.127 | 0.873 |
| 165 | BOM3 (0): | 0.353 | 0.647 |
| 166 | BOM4 (0): | 0.454 | 0.546 |
| 167 | BOM5 (0): | 0.957 | 0.043 |
| 168 | BOM6 (0): | 0.446 | 0.554 |
| 169 | BOM7 (0): | 0.978 | 0.022 |
| 170 | TAR10 (0): | 0.982 | 0.018 |
| 171 | TAR11 (0): | 0.954 | 0.046 |
| 172 | TAR12 (0): | 0.921 | 0.079 |
| 173 | TAR13 (0): | 0.911 | 0.089 |
| 174 | TAR14 (0): | 0.947 | 0.053 |
| 175 | TAR15 (0): | 0.971 | 0.029 |
| 176 | TAR16 (0): | 0.972 | 0.028 |
| 177 | TAR1 (0): | 0.980 | 0.02 |
| 178 | TAR2 (0): | 0.979 | 0.021 |
| 179 | TAR3 (0): | 0.981 | 0.019 |
| 180 | TAR4 (0): | 0.947 | 0.053 |
| 181 | TAR5 (0): | 0.988 | 0.012 |
| 182 | TAR6(10): | 0.807 | 0.193 |
| 183 | TAR7 (0): | 0.988 | 0.012 |
| 184 | TAR8(10): | 0.986 | 0.014 |
| 185 | WAL1 (5): | 0.974 | 0.026 |
| 186 | WAL2 (0): | 0.986 | 0.014 |
| 187 | BELF10 (0): | 0.314 | 0.686 |
| 188 | BELF11 (0): | 0.361 | 0.639 |
| 189 | BELF12 (5): | 0.402 | 0.598 |
| 190 | BELF13 (5): | 0.137 | 0.863 |
| 191 | BELF17 (0): | 0.728 | 0.272 |
| 192 | BELF18 (0): | 0.974 | 0.026 |
| 193 | BELF19(10): | 0.933 | 0.067 |
| 194 | BELF20 (0): | 0.647 | 0.353 |
| 195 | BELF21 (0): | 0.883 | 0.117 |
| 196 | BELF22 (0): | 0.707 | 0.293 |
| 197 | BELF2-20: | 0.471 | 0.529 |
| 198 | BELF3 (0): | 0.740 | 0.26 |
| 199 | BELF4 (5): | 0.333 | 0.667 |
| 200 | BELF5 (5): | 0.949 | 0.051 |
| 201 | BELF6 (0): | 0.946 | 0.054 |
| 202 | BELF9 (0): | 0.979 | 0.021 |
| 203 | MILL10 (0): | 0.289 | 0.711 |
| 204 | MILL12 (0): | 0.948 | 0.052 |
| 205 | MILL14 (0): | 0.884 | 0.116 |
| 206 | MILL16 (0): | 0.986 | 0.014 |
| 207 | MILL17 (0): | 0.715 | 0.285 |
| 208 | MILL18 (0): | 0.193 | 0.807 |
| 209 | MILL19 (5): | 0.502 | 0.498 |
| 210 | MILL1 (0): | 0.627 | 0.373 |
| 211 | MILL20 (0): | 0.878 | 0.122 |
| 212 | MILL21 (0): | 0.937 | 0.063 |
| 213 | MILL3(10): | 0.697 | 0.303 |
| 214 | MILL4 (0): | 0.984 | 0.016 |
| 215 | MILL5 (0): | 0.692 | 0.308 |
| 216 | MILL6 (0): | 0.964 | 0.036 |
| 217 | MILL8 (0): | 0.934 | 0.066 |
| 218 | MILL9 (0): | 0.247 | 0.753 |
| 219 | SIN10 (0): | 0.979 | 0.021 |
| 220 | SIN13(10): | 0.854 | 0.146 |
| 221 | SIN14 (5): | 0.429 | 0.571 |
| 222 | SIN2 (0): | 0.902 | 0.098 |
| 223 | SIN3 (0): | 0.906 | 0.094 |
| 224 | SIN4 (0): | 0.506 | 0.494 |
| 225 | SIN5(10): | 0.984 | 0.016 |
| 226 | SIN7 (0): | 0.243 | 0.757 |
| 227 | SIN8 (0): | 0.128 | 0.872 |
| 228 | PUTTY1-15: | 0.938 | 0.062 |
| 229 | PUTTY3 (0): | 0.982 | 0.018 |
| 230 | PUTTY4 (0): | 0.985 | 0.015 |
| 231 | PUTTY5(10): | 0.955 | 0.045 |
| 232 | PUTTY6 (5): | 0.971 | 0.029 |
| 233 | PUTTY7 (0): | 0.869 | 0.131 |
| 234 | SCY1 (0): | 0.218 | 0.782 |
| 235 | SCY2 (0): | 0.884 | 0.116 |
| 236 | SCY3 (0): | 0.770 | 0.23 |
| 237 | KNU1 (0): | 0.844 | 0.156 |
| 238 | KNU2 (0): | 0.331 | 0.669 |
| 239 | FAI2 (0): | 0.895 | 0.105 |
| 240 | FAI6 (0): | 0.977 | 0.023 |
| 241 | FAI7 (0): | 0.897 | 0.103 |
| 242 | LIB10 (0): | 0.955 | 0.045 |
| 243 | LIB12 (0): | 0.911 | 0.089 |
| 244 | LIB2 (0): | 0.978 | 0.022 |
| 245 | LIB3 (0): | 0.974 | 0.026 |
| 246 | LIB4 (0): | 0.955 | 0.045 |
| 247 | LIB5 (0): | 0.982 | 0.018 |
| 248 | LUD1 (0): | 0.981 | 0.019 |
| 249 | LUD2 (0): | 0.953 | 0.047 |
| 250 | LUD3 (0): | 0.975 | 0.025 |
| 251 | ING1 (5): | 0.851 | 0.149 |
| 252 | ING2 (0): | 0.966 | 0.034 |
| 253 | ING3 (5): | 0.971 | 0.029 |
| 254 | MANT1(10): | 0.125 | 0.875 |
| 255 | MANT2 (0): | 0.980 | 0.02 |
| 256 | MANT3 (0): | 0.979 | 0.021 |
| 257 | MANT4 (0): | 0.916 | 0.084 |
| 258 | MANT5 (0): | 0.944 | 0.056 |
| 259 | MANT6 (0): | 0.986 | 0.014 |
| 260 | NOWRA10 (0): | 0.917 | 0.083 |
| 261 | NOWRA11 (0): | 0.855 | 0.145 |
| 262 | NOWRA13(10): | 0.944 | 0.056 |
| 263 | NOWRA21 (0): | 0.244 | 0.756 |
| 264 | NOWRA3 (0): | 0.339 | 0.661 |
| 265 | NOWRA4(10): | 0.948 | 0.052 |
| 266 | NOWRA5 (0): | 0.356 | 0.644 |
| 267 | NOWRA6 (0): | 0.942 | 0.058 |
| 268 | NOWRA7(10): | 0.047 | 0.953 |


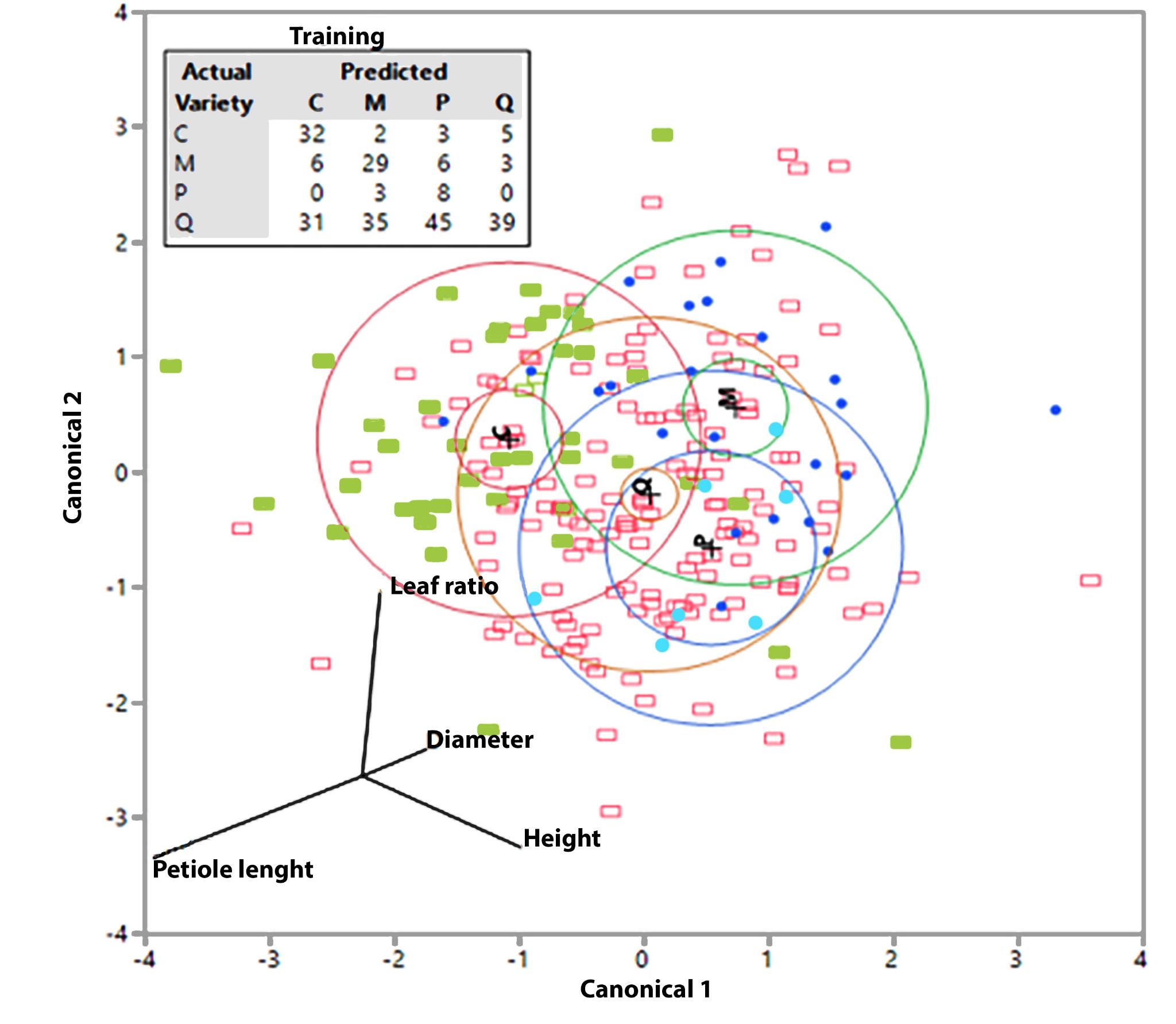


**Figure S1.** Leaf morphology in 18-month seedlings of *Eucalyptus moluccana,* subspecies *crassifolia* (C, green squares/red circles), *queenslandica* (Q, red squares/orange circles), *pedicellata* (P, turquoise dots/blue circles) and *moluccana* (M, blue dots/green circles). Internal circle depicts the 95% confidence region for the means on the canonical variables of the group and the external circle denotes the space of the first two canonical variables that contains approximately 50% of the observations. The analysis included 247 samples of which 139 (56%) was misclassified.


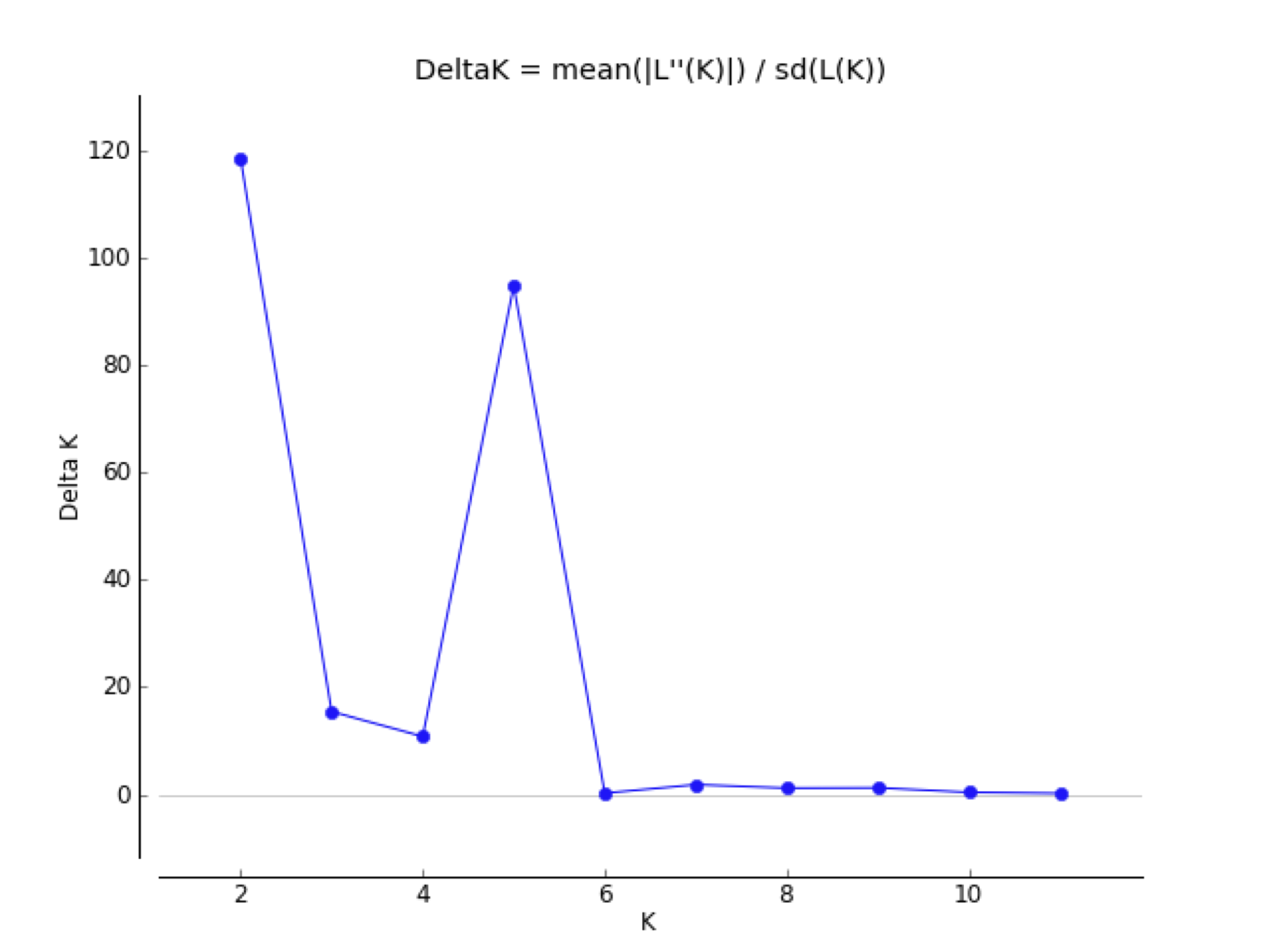


**Figure S2.** Structure analysis of Grey box *Eucalyptus moluccana* was done with 10 microsatellites in 268 individuals, 29 localities. A) Evanno test suggesting two regions (K=2) as per the highest DeltaK associated with the two populations, followed by K=5.


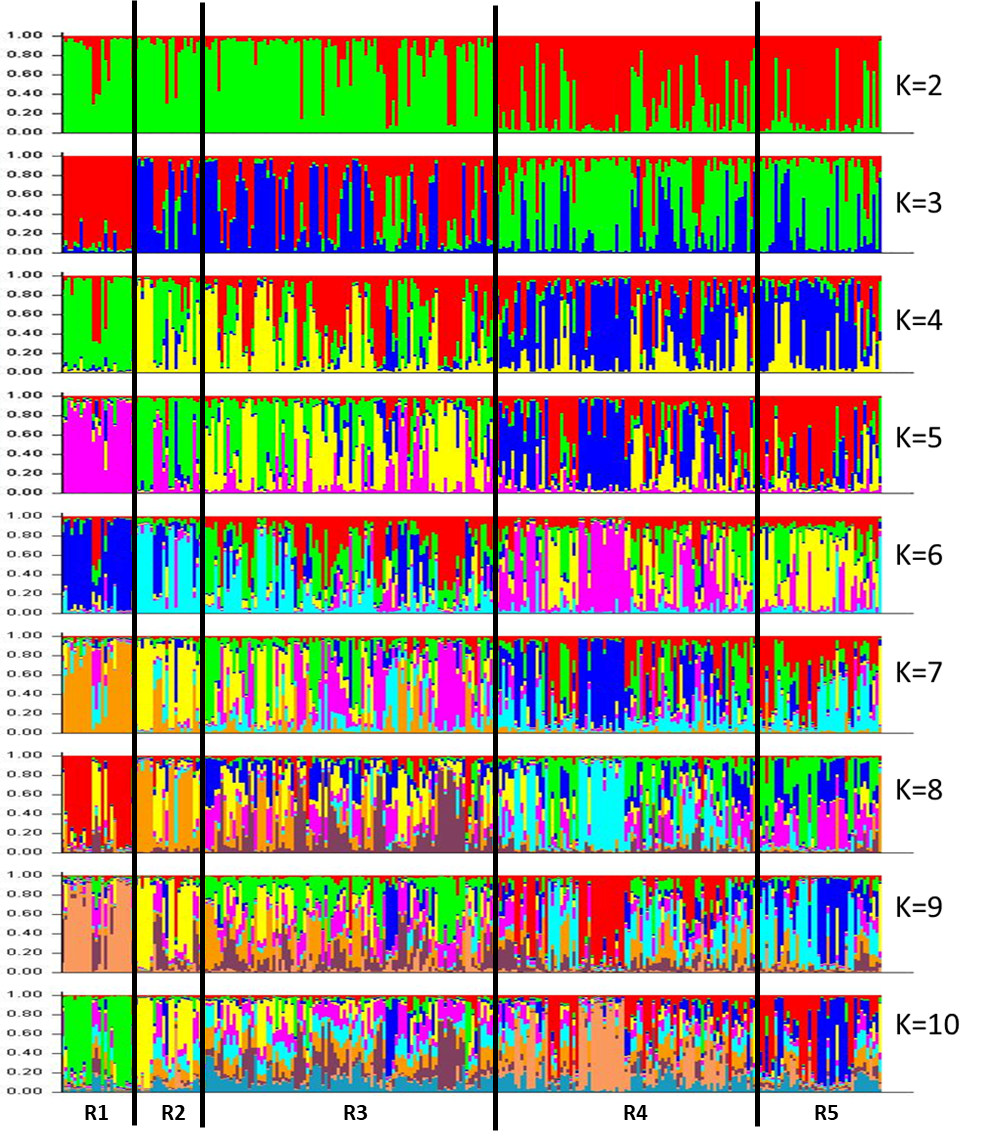


**Figure S3.** Structure analysis including 10 polymorphic microsatellites of 268 individuals from 29 populations of *E. moluccana* across its whole distribution ranging from K=2 to K=10.


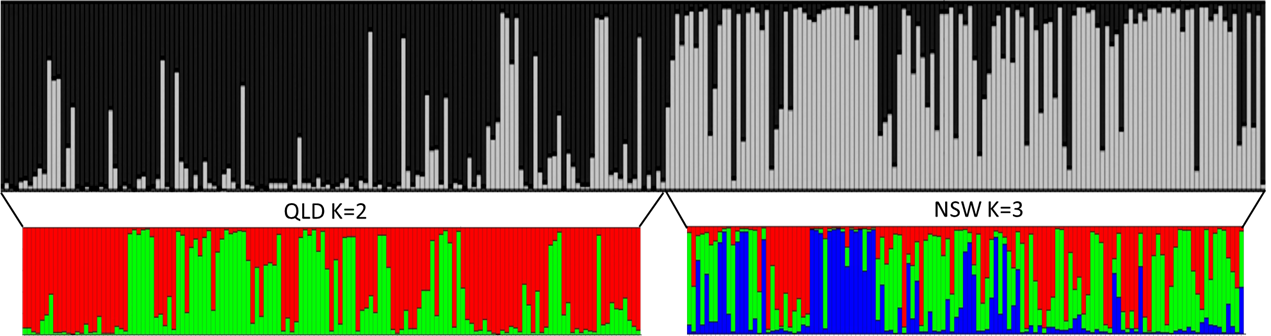


Figure S4. Substructuring approach showing initial K=2 and further substructuring the northern populations (QLD) in K=2 and the southern population (NSW) in K=3.

**Figure S5.** PCoA of 268 individuals of *E. moluccana* using 10 SSRs. First axis explains 5.13% of the variation, and the second axis explains 4.18% of the variation. Samples were colored based on their region of origin (see Figure 2 in the main text).
